# Motor Evoked Potentials as Potential Biomarkers of Early Atypical Corticospinal Tract Development in Infants with Perinatal Stroke

**DOI:** 10.3390/jcm8081208

**Published:** 2019-08-13

**Authors:** Jesse L. Kowalski, Samuel T. Nemanich, Tanjila Nawshin, Mo Chen, Colleen Peyton, Elizabeth Zorn, Marie Hickey, Raghavendra Rao, Michael Georgieff, Kyle Rudser, Bernadette T. Gillick

**Affiliations:** 1 Department of Rehabilitation Medicine, University of Minnesota, Minneapolis, MN 55455, USA; 2 Department of Psychiatry, University of Minnesota, Minneapolis, MN 55455, USA; 3 Department of Physical Therapy and Human Movement Sciences, Northwestern University, Chicago, IL 60611, USA; 4 Division of Neonatology, Children’s Hospitals and Clinics of Minnesota, Minneapolis, MN 55455, USA; 5 Department of Pediatrics, University of Minnesota, Minneapolis, MN 55455, USA; 6 Division of Biostatistics, University of Minnesota, Minneapolis, MN 55455, USA

**Keywords:** perinatal stroke, cerebral palsy, transcranial magnetic stimulation, infant, pediatrics, non-invasive brain stimulation

## Abstract

Diagnosis of cerebral palsy (CP) after perinatal stroke is often delayed beyond infancy, a period of rapid neuromotor development with heightened potential for rehabilitation. This study sought to assess whether the presence or absence of motor evoked potentials (MEPs) elicited by transcranial magnetic stimulation (TMS) could be an early biomarker of atypical development within the first year of life. In 10 infants with perinatal stroke, motor outcome was assessed with a standardized movement assessment. Single-pulse TMS was utilized to assess presence of MEPs. Younger infants (3–6 months CA, *n* = 5, 4/5 (80%)) were more likely to present with an MEP from the more-affected hemisphere (MAH) compared to older infants (7–12 months CA, *n* = 5, 0/5, (0%)) (*p* = 0.048). Atypical movement was demonstrated in the majority of infants with an absent MEP from the MAH (5/6, 83%) compared to those with a present MEP (1/4, 25%) (*p* = 0.191). We found that age influences the ability to elicit an MEP from the MAH, and motor outcome may be related to MAH MEP absence. Assessment of MEPs in conjunction with current practice of neuroimaging and motor assessments could promote early detection and intervention in infants at risk of CP.

## 1. Introduction

Perinatal stroke, occurring between 20 weeks’ gestation and the 28th day of life, often leads to cerebral palsy (CP), a diagnosis of disordered movement due to a disturbance to the developing brain in utero or infancy [1,2,3]. CP is the leading cause of childhood motor disability with estimates of worldwide prevalence of CP as high as 4 in 1000 live births [4,5]. The development of the primary motor pathway for volitional movement, the corticospinal tract (CST), may be impacted after perinatal stroke, resulting in motor impairment [6,7]. Little is known about the time at which maladaptive neurodevelopment of the CST occurs after perinatal stroke, or its relationship with motor function within the first year of life and CP diagnosis. An estimated 68% of infants with perinatal stroke will eventually be diagnosed with CP, but the diagnosis of CP is often delayed until notable movement discrepancies become apparent, often at two years of age or greater [8,9]. Delay of diagnosis defers rehabilitation services and prevents intervention during the first year of life, an influential and highly neuroplastic period of development [10,11,12]. We hypothesize that identification of neural biomarkers of atypical CST development in infants with perinatal stroke at risk of CP could be achieved through non-invasive brain assessments with transcranial magnetic stimulation (TMS).

TMS is a form of electromagnetic non-invasive brain stimulation which can assess cortical excitability and CST organization through depolarization of corticospinal neurons [13]. A known biomarker of hemiparetic CP is the absence of TMS-induced motor evoked potentials (MEPs) from the more-affected hemisphere (MAH) [14,15,16,17]. Absence of MEPs from the MAH is representative of atypical CST organization and correlates with poorer hand function in children with hemiparetic CP [14,15,16,17]. TMS assessment of cortical excitability in infants with perinatal stroke could inform whether neural biomarkers commonly present in older children with CP can be detected in infancy prior to behavioral manifestation of motor deficits. While early identification of CP can be reliably achieved through the combination of neuroimaging and motor assessments, TMS may contribute additional diagnostic and prognostic information [18]. For example, though approximately 90% of children with CP have atypical neuroimaging findings, a remaining 10% display typical findings which potentially impedes early diagnosis [19]. If identified in infancy, the absence of an MEP from the MAH may elucidate the timing and mechanism of atypical CST organization, as well as potential prognosis of anticipated functional outcomes based upon known MEP and motor outcomes in older children with CP. Early identification of biomarkers of atypical CST organization in infants at risk of CP would facilitate early intervention after perinatal stroke during a highly influential period of development to promote optimal formation of neuromotor systems. The purpose of this pilot study was to identify whether the absence of MEPs in infants with perinatal stroke is related to: (1) age and (2) motor outcome.

## 2. Methods

### 2.1. Participants

This cross-sectional pilot study was approved by the University of Minnesota’s (UMN) Institutional Review Board, Fairview Health Services, the Clinical and Translational Sciences Institute, Center for Magnetic Resonance Research (CMRR), and the UMN Discovery, Research, and Innovation Economy initiative steering committee. Infants were recruited from the University of Minnesota’s Masonic Children’s Hospital (UMMCH) neonatal intensive care unit (NICU) and the Children’s Hemiplegia and Stroke Association. Infants between 3–12 months of age corrected for prematurity, with a diagnosis of unilateral or asymmetric bilateral perinatal stroke confirmed by cranial ultrasound or magnetic resonance imaging (MRI), were included. Infants with MRI incompatible implanted medical devices, uncontrolled seizure, other neurological diagnoses, genetic disorders, or neoplasm were excluded. A total of 10 infants meeting criteria enrolled and participated. Parents provided informed consent for their infants to participate in this study. Data collection occurred at UMN and UMMCH between August 2016 and January 2018.

### 2.2. Movement Assessment and Motor Outcome

Age-appropriate standardized behavorial assessments were completed to examine motor outcome. Dependent on the postnatal corrected age (CA) for prematurity of the child, either the Prechtl General Movements Assessment (GMA) or Bayley Scales of Infant and Toddler Development III (BSID-III) was completed. The GMA was utlized for infants 3–5 months CA, and consisted of a 3–5 *min* video of the infant naturally moving while lying supine with clearly visible upper and lower extremities. GMA videos were taken by 20 weeks CA at home by a parent or by a member of the research team in the UMMCH NICU follow-up clinic. All GMA videos were scored by the same GMA-certified pediatric physical therapist (C.P.). The BSID-III motor scale, which consisted of fine and gross motor subsets, was utilized for infants 6–12 months CA and was administered and scored by the same pediatric physical therapist (J.L.K). Gestational age (GA) and CA were determined by Academy of Pediatrics definitions, and medical record review [20]. Level of prematurity was determined by GA and World Health Organization definitions of extremely perterm (<28 weeks GA), very preterm (28–31 weeks GA), moderate to late preterm (32–36 weeks GA), and term (37–41 weeks GA) [21].

### 2.3. Transcranial Magnetic Stimulation

As a means to guide TMS assessment, magnetic resonance imaging (MRI) sequences were collected at the CMRR at UMN per our published study protocol, which utilized procedures from the Baby Connectome Project for infant scanning techniques [22,23]. Participants’ T1-weighted anatomical MRIs were utilized to reconstruct a 3D brain model for stereotactic neuronavigated guidance of TMS coil placement (Brainsight, Rogue Research, Montreal, Quebec, Canada). The hand knob region of the primary motor cortex (M1) corresponding to the area of the wrist flexors for each hemisphere was visually identified and labeled on participants’ 3D brain models in the Brainsight software for guidance of TMS pulse delivery. A lightweight (4.9 g) 3D printed head-tracker was attached to the forehead with sensitive skin tape. Landmarks of nasion, tip of nose, and left and right pre-auricular notches were utilized to register and synchronize the 3D brain models to the participants within the Brainsight software for realtime motion tracking of the TMS coil in relation to the identified M1 target for each infant.

Muscle responses to TMS were recorded via surface electromyography (EMG) of the bilateral wrist flexors. The wrist flexors were selected as the optimal muscle group for EMG recording due to pilot testing results which demonstrated robust resting EMG amplitude and lack of cardiac artifact present in signal from the biceps bracii [22]. The skin was prepped and cleaned, and stainless steel surface EMG electrodes (101085, Natus Neurology Inc., Pleasanton, CA, USA) were placed on the ventral forearm over the wrist flexor muscle belly and secured with tape and coban. Ground electrodes (TD-431, Discount Disposables, Mansfield Research and Development LLC, St. Albans, VT, USA) were placed over the bilateral olecranon processes. EMG signals were amplified (Y03-2, Motion Lab System, Inc., Baton Rouge, LA, USA) with the gain of ×300, filtered (band-passed with 15 to 2000 Hz) and digitalized (NI 9234, National Instruments, Austin, TX, USA) at 6.4k Hz for offline analysis. All data were collected using a custom LabVIEW program (v2012, National Instruments, Austin, TX, USA).

Single-pulse TMS was delivered to the targeted M1 with a 70 mm figure-of-eight coil (Remote Double, Magstim Company Ltd., Carmanthenshire, UK) connected to a TMS stimulator (Magstim Bistim^2^ and 200^2^ unit set, Magstim Co. Ltd., Carmanthenshire, UK) placed on the scalp at a 45 degree angle posteriorlateral to the midsagittal plane. Dependent upon the postural control and preference of the infant, infants were positioned supported in their parent’s lap, or independently seated or standing. When possible, testing was completed with the infants’ upper extremities at rest through activities such as passive bottle feeding or watching a video to achieve a relaxed state. TMS was first delivered to the targeted M1 of the left hemisphere beginning at 50–60% maximum stimulator output (MSO) and then increased by 5% increments after 3–5 trials at each MSO with a pre-determined ceiling of 90% MSO (average number of trials = 20, range of trials (6–47 trials); range of TMS intensities (50–90% MSO)). If 90% MSO was attained and no potential MEP responses were identified, the surrounding cortex was assessed in anterior, posterior, medial, and lateral directions by moving the coil approximately 0.5 cm in each direction with 2–3 trials at each new location. The right hemisphere was then assessed following the same protocol (average number of trials = 20, range of trials (8–46 trials); range of TMS intensities (50–90% MSO)). Number of trials and intensities completed were dependent upon infant tolerance and time limitations.

**Figure 1:**
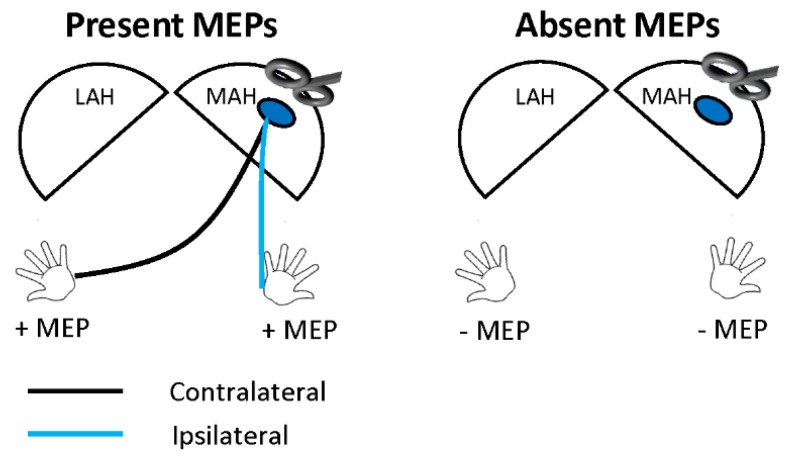
Example of transcranial magnetic stimulation assessment of motor evoked potentials (MEPs) from the more-affected hemisphere (MAH). Presence of MEPs (+MEP) was defined by presence of at least one MEP in either the contralateral (black line), ipsilateral (blue line), or both wrist flexors. MEP absence (−MEP) was defined by lack of MEP presence in the wrist flexor of either arm.

EMG signals were post-processed in MATLAB software (v2018b, Mathworks, Inc. Natick, MA, USA). The raw EMG signal was baseline corrected and rectified. The area under the curve (AUC) of each MEP and pre-stimulus activity was cacluated as defined by Rossini et al. [13]. A 10 ms pre-stimulus period of −13 to −3 ms was selected for all trials to quantify the EMG signal direcly prior to the TMS pulse exluding the stimulus artifact in the pre-stimulus AUC. Post-processed EMG signals from the right and left wrist flexors were visually assessed for motor evoked potentials (MEPs) occuring within 15–50 ms after TMS onset. Within this latency, an MEP was defined as ‘present’ by the demonstration of a peak-to-peak amplitude of at least 50 µV, greater than 120% of the standard deviation (SD) of pre-stimulus EMG amplitude, and an MEP AUC greater than pre-stimulus AUC (Supplementary Materials, Figure S1). For each hemisphere, MEPs were classified as present (+MEP) if an MEP was recorded in either wrist flexor, and absent (−MEP) if no MEP was attained from either wrist flexor (Figure 1).

### 2.4. Safety

Medical records were screened for eligibility criteria and reviewed by the research and medical teams prior to participant enrollment. The Medical Director determined final eligibility of all infants. Assessments of heart and respiratory rate, blood pressure, behavioral state, and stress responses were completed prior to commencing and after completing TMS assessments. Ear plugs were utilized to protect the infants’ hearing during TMS assessments. Assessment of behavioral state and stress responses were completed every 5 *min* throughout the TMS assessments. A member of the research team followed up by phone with parents of participating infants 24 *h* after TMS assessment to identify any potential adverse events or behavioral changes. The Medical Monitor reviewed the documentation upon completion of the study for each infant, and if needed, evaluated any potential adverse events to determine optimal recommendations for care.

### 2.5. Statistical Analysis

Data are presented as frequency counts and percentages, and descriptive statistics of mean, SD, and range. Proportions of groups were compared using Fisher’s exact tests. MEP responses from the MAH and less-affected hemisphere (LAH) were compared across groups defined by CA and GA. Motor outcome was compared between those with and without MEP responses within each hemisphere. For analysis, infants were grouped as younger infants (3–6 months CA) or older infants (7–12 months CA) and by GA as preterm (GA < 37 weeks) or term (GA ≥ 37 weeks). Infants were also categorized as having atypical or typical movement as determined by GMA or BSID-III. Motor outcomes (atypical or typical movement) were compared to variables of CA (younger or older) and GA (preterm or term). All statistical analyses were completed using *R* version 3.4.1 (The *R* Foundation for Statistical Computing, Vienna, Austria).

## 3. Results

### 3.1. Safety of Transcranial Magnetic Stimulation

The infants’ tolerance to TMS was determined based on previously reported safety assessments, follow up calls with no adverse events reported by parents 24 *h* post-TMS assesssment, and no indications or adverse events for Medical Monitor review [24]. All vital signs acquired at pre- and post-TMS assessment were within age dependent normative ranges [24,25].

### 3.2. Participant Characteristics

The mean GA of participating infants was 31 weeks (*SD* = 8 weeks, range: 22–41 weeks), and mean CA at time of TMS assessment was 7 months (*SD* = 3 months, range: 3–12 months). The majority of infants were male (70%) and born preterm (70%) (Table 1). In 60% of participants, the left hemisphere was more-affected.

### 3.3. Motor Evoked Potentials and Age

**Figure 2:**
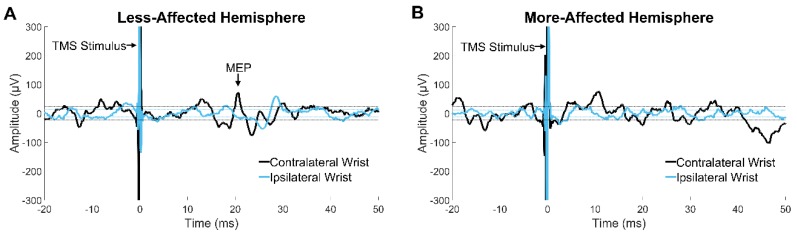
Surface electromyography (EMG) recording from the contralateral (black line) and ipsilateral (blue line) wrist flexors in a 12-month-old infant with unilateral perinatal stroke. (**A**) Motor evoked potential (MEP) in the contralateral wrist flexor elicited by transcranial magnetic stimulation (TMS) of the less-affected hemisphere. (**B**) EMG signal displaying absence of MEP in either wrist flexor after TMS of the more-affected hemisphere. Both traces A and B were recorded at a TMS intensity of 85% of maximum stimulator output.

TMS of the MAH revealed primarily absent MEPs from the bilateral wrist flexors (6/10 infants; 60%) (Figure 2). A statistically significant difference in MEP presence from the MAH was identified based on CA group (*p* = 0.048) with 4/5 younger infants (80%) and 0/5 older infants (0%) displaying MEPs from the MAH (Table 2). No significant difference in MEP presence from the LAH was identified, though the younger group had the highest percentage consistent with responses from the MAH, with 5/5 younger infants (100%) and 3/5 older infants (60%) displaying MEPs from the LAH (*p* = 0.444). MEP presence based on GA group was not significantly different for the MAH (3/7 (43%) preterm and 1/3 (33%) term; *p* = 1) or LAH (6/7 (86%) preterm and 2/3 (67%) term; *p* = 1). In total, 4/10 infants (40%) displayed MEPs from the MAH and 8/10 infants (80%) displayed MEPs from the LAH (Table 3).

### 3.4. Motor Evoked Potentials and Motor Outcomes

In 5/8 infants with MEPs, the mean pre-stimulus AUC was lower in +MEP trials compared to –MEP trials (Supplementary Materials, Table S1, Figure S2). As determined by GMA and BSID-III assessments, atypical movement was observed in 60% of all infants (*n* = 10). Though not statistically significant, the proportion of infants with atypical movement was higher among those without MEP response compared to those with MEP from the MAH (5/6 −MEP (83%), 1/4 +MEP (25%); *p* = 0.191) and the LAH (2/2 −MEP (100%), 4/8 +MEP (50%); *p* = 0.467) (Table 4). Atypical movement was observed in 40% of younger infants (2/5) and 80% of older infants (4/5) with no significant difference in motor outcome based on CA group (*p* = 0.524). No significant difference in motor outcome was determined based on GA group comparison (*p* = 1) with 4/7 preterm (57%) and 2/3 term infants (67%) displaying atypical movement.

## 4. Discussion

As a means to determine whether MEP absence may be an early biomarker of atypical CST development within the first year of life, we assessed the prevalence of atypical movement and MEP responses to TMS in 10 infants with perinatal stroke. Here we explore the results of our aims to investigate the relationship between MEP presence and (1) age and (2) motor outcome.

### 4.1. Relationship between MEP Presence and Age

A significant difference in MEP outcome was identified based on corrected age, with MEPs from the MAH present in 4/5 younger infants (3–6 months CA) and 0/5 older infants (7–12 months CA). Our results suggest that younger infants with perinatal stroke are more likely to display TMS elicited MEPs from the MAH. Interestingly, none of the older infants displayed presence of an MEP from stimulation of the MAH, signifying an early age-related neurophysiologic difference in infants with perinatal stroke. Our findings are synonymous with Eyre et al.’s report of TMS outcomes of absence of MEPs from stimulation of the injured hemisphere of infants with unilateral perinatal stroke after 12 months of age [6]. In their prior study utilizing single-pulse TMS to compare infants with perinatal stroke and typical development, both contralateral and ipsilateral MEPs were elicited from both hemispheres until 3 months of age in all infants [6]. In infants with unilateral perinatal stroke, MEPs from stimulation of the injured hemisphere were lost by 12 months of age [6]. In typically developing infants, ipsilateral MEPs continued to be elicited, but decreased in amplitude in comparison to the contralateral MEPs by 6 months of age, suggesting development of a predominantly contralateral pattern of CST organization occurs around 6 months of age [6]. Neuroimaging studies have also reported detectable changes in the CST within the first year of life in infants with unilateral perinatal stroke, with lower CST volumes of the injured hemisphere compared to the non-injured hemisphere [6,14]. Asymmetric CST volumes in infancy were predictive of later MEP absence from the injured hemisphere in children with CP, establishing the existence of a relationship between CST structure and neurophysiologic outcomes measured by TMS [14]. These previously reported findings, in addition to our own, suggest that the first 6 months of life are a formative period of CST development, with early atypical CST development in infants with perinatal stroke becoming evident between 7–12 months of age [6]. Further TMS investigations with larger samples of infants with perinatal stroke at risk of CP may elucidate additional potential differences in MEP laterality, latency, amplitude, frequency, and MSO required to elicit a response to determine whether subtle changes in CST development are evident within the first 6 months of life. Investigation of these additional variables could guide determination of the earliest age at which TMS could be utilized to identify biomarkers of atypical CST development in infants at risk of CP.

A relationship between gestational age and MEP presence was not determined, with preterm and term infants displaying no significant differences in MEP outcomes. No known prior TMS studies have investigated the relationship of gestational age and MEP presence in infants with perinatal stroke. However, one study in older children with CP determined preterm or term birth did not appear to influence MEP outcomes or motor performance [14]. In contrast, a systematic review identified 10 studies reporting low gestational age to be positively associated with CP diagnosis [26], which suggests further investigation of the impact of gestational age on MEP presence may be warranted in infants at risk of CP.

### 4.2. Relationship between MEP Presence and Motor Outcome

A strong trend of atypical movement in 83% of infants with absence of MEPs from the MAH compared to 25% with present MEPs suggests a relationship between cortical excitability and motor outcome may exist in infants with perinatal stroke at risk of CP. Eyre et al. reported similar findings in infants with unilateral perinatal stroke, with absence of MEPs from the injured hemisphere being associated with failure to display a grasp by two years of age [6]. Though ours is the first study, to our knowledge, to compare TMS results with motor outcome in the first year of life, similar trends of MEP absence and atypical movement are demonstrated in TMS literature in older children and adolescents with CP. Four studies of TMS and motor outcomes in children with CP due to unilateral perinatal stroke reported an association between hand function and CST organization, with poorer hand function demonstrated by children lacking MEPs from the injured hemisphere [14,15,16,17]. CST volume asymmetry in infants with unilateral perinatal stroke has been reported to be predictive of CP diagnosis, poorer hand function, and MEP absence from the injured hemisphere later in childhood and adolescence, thus identifying a relationship between early CST differences and later motor outcome [14]. These findings support the efficacy of MEP absence from the MAH as a biomarker of atypical CST organization and motor function in older children with CP, and highlight the value of exploring early detection of this biomarker after perinatal stroke.

No consistent trends were demonstrated regarding amount of pre-stimulus activity and MEP presence in infants. The mean pre-stimulus AUC was lower in +MEP trials compared to −MEP trials in 5/8 infants with MEPs, contradictory to reports that higher pre-stimulus muscle activity facilitates MEP elicitation in older children and adults [13]. MEPs were obtained from stimulation of the LAH in 8/10 infants with perinatal stroke ranging from 3–12 months CA, whereas only 4/10 infants displayed MEPs from stimulation of the MAH. Our results suggest that infants with perinatal stroke display early differences in TMS elicited MEPs in the wrist flexors from the more and less-affected hemispheres. The phenomenon of absence of MEPs in the first year of life may be unique to perinatal stroke as infants with brachial plexus injury [27], epilepsy [28], and typically developing infants [6] displayed intact MEPs in the upper extremity musculature. As such, the absence of MEPs from the MAH could be a unique early biomarker of atypical neurodevelopment of the CST in infants with perinatal stroke at risk of CP.

### 4.3. Clinical Relevance

Absence of MEPs from the MAH is a demonstrated biomarker of atypical CST organization in older children with CP [14,15,16,17] and may be detected in infants with perinatal stroke as young as 5 months of age, with infants 7–12 months of age consistently displaying MEP absence from the MAH. The use of TMS to identify early biomarkers of atypical CST development in conjunction with the current gold standard of neuroimaging and motor assessments could contribute to earlier identification of infants at risk of CP [18]. Early identification of risk of CP within the first year of life after perinatal stroke is crucial for facilitation of more timely rehabilitation to take advantage of the period of rapid neuromotor development with the goal of more complete recovery of motor function and prevention of motor disability.

Myelination and synaptogenesis begin in the last trimester and continue throughout childhood as the brain continues to mature, with the first three years of life being an especially prolific period of neural development [29]. The formation of neural networks and pathways in the developing brain incorporates the concept of Hebbian plasticity—the strengthening of synapses with repetitive synchronous firing of pre- and postsynaptic neurons [30]. Early experiences and interactions with environmental stimuli result in the strengthening or pruning of synapses via Hebbian plasticity, thus shaping neurodevelopment [30]. Based on our results of MEP presence related to corrected age, it is possible that experience-dependent plasticity drives CST connectivity and maturation, and that a sensitive period exists within the first 6 months of life which influences structural and functional outcomes later in development [30]. Sensitive periods of development are known to exist in many mammalian systems including the motor, somatosensory, visual, auditory, communication, gustatory, and olfactory systems [31]. Evidence also confirms that early life sensory experiences during sensitive periods strongly influence synaptogenesis and neurodevelopment and shape long-term structural and functional outcomes [31]. Inadequate experience-dependent neuronal stimulation and weaker signaling strength of corticospinal neurons from the MAH during a formative period of development may lead to the pruning of weaker downstream neuromuscular junction synapses and result in atypical neuromuscular innervation and movement patterns [32,33,34,35]. Such experience-dependent mechanisms of neuroplasticity could underlie atypical CST development after perinatal stroke, resulting in loss of MEPs from the MAH and impaired motor function.

Identification of a potential sensitive period for experience-dependent plasticity of the CST could inform optimal timing for intervention. Given our findings that infants 6 months of age or less were more likely to demonstrate presence of MEPs elicited from the MAH, rehabilitation with the first 6 months of life may have the greatest impact on optimal CST organization. In a rodent stroke model, rehabilitation interventions harnessing experience-dependent plasticity have been shown to increase synaptic density in layer V pyramidal neurons in the motor cortex through training in an upper limb reaching task [36]. The increase in density of pyramidal neuron synapses correlated with improved motor recovery outcomes, demonstrating the influence of motor training on neuronal structure and motor function after stroke [36]. Viable CST connections were still present in 4/5 infants 6 months of age or less, suggesting that early rehabilitation interventions targeting the strengthening of those neural pathways within the first 6 months of life could promote optimal development of the CST, which is known to correlate to motor function [14,15,16,17]. Longitudinal investigation of CST connectivity after perinatal stroke could better identify the critical window and mechanisms of CST development to inform the most impactful rehabilitation timing and strategies. Greater knowledge of neurodevelopmental changes after perinatal stroke will drive continued advances in implementation of best practice strategies and maximization of the functional potential of children with CP throughout their lifespan.

### 4.4. Limitations

Though a trend of MEP absence was identified in infants with atypical movement, our small sample size and the heterogeneity of our sample limits our ability to determine whether statistically significant differences exist. The heterogeneous diagnosis of perinatal stroke affects a variety of brain areas and results in variable clinical presentations. As such, it is possible that lesion location and the unilateral or bilateral nature of the lesion may influence TMS and motor outcomes. 

The assessment of MEPs with established protocols, such as resting motor threshold, is not feasible in infants as they cannot remain consistently at rest or complete repeated contractions at a constant force, resulting in deviation from typical TMS protocols and MEP analysis. Our protocol utilized concurrent activities to encourage relaxation of the upper extremities, such as bottle feeding or watching a video, to attempt to facilitate consistent resting muscle activity during TMS testing. MEPs were analyzed with a requirement of an amplitude greater than 120% of the variance of pre-stimulus activity to overcome variability in fluctuating levels of muscle activity. Latencies for TMS elicited MEPs are yet unknown in infants, thus we included MEPs occurring within 15–50 ms after the TMS stimulus given consistent patterns of post-stimulus EMG activity in our data. Infants may display different neural conduction velocities from older children and adults due to still developing myelination [29,37]. In the two infants in whom we were unable to record MEPs from either hemisphere, it is possible that the location targeted for stimulation was not the true anatomical area of M1 associated with motor control of the wrist flexors in those infants, and the assessment of the surrounding cortex was not completed in a wide enough radius. TMS assessment time and number of trials was limited by infant compliance and tolerance, and a comprehensive systematic evaluation of the cortical area of interest utilizing standard protocols implemented in older children and adults is not feasible in infants [24,27,38]. Given prior reports of motor thresholds being greatest within the first year of life, and higher in infants with perinatal stroke compared to typically developing infants [6], these two infants may have demonstrated a motor threshold which exceeded TMS intensities of 90% MSO, which was the highest intensity we utilized. With the machine we used, it is possible that stimulation up to 100% MSO could have elicited MEPs in infants at which an MEP was not elicited in either hemisphere at 90% MSO. The variability in TMS intensity required to elicit a response may also be related to the type of stimulator utilized, as well as the electric field generated in individual infant brains (e.g., due to brain size, variation in anatomy, lesions, and overall conductivity). For the specific stimulator utilized in this study, the rate of delivery of current at 100% was 186 Amps per microsecond or 186 A/uS. The range of 50–90% MSO utilized would then equate to a delivery rate for this unit of 93.0–167.4 A/uS. Rates between stimulators could vary, and should be considered in future studies for reproducibility and comparison. Regardless, our findings support the existence of an age-related significant difference in cortical excitability of the MAH. Investigations of the variability in overall energy required to elicit a detectable difference in cortical excitability would be beneficial.

An analysis of CP diagnosis outcome in our full sample of 10 infants was not possible at this time due to variable length of follow-up and inconsistent identification of diagnosis (definition and timing). Physicians commonly wait to provide a diagnosis of CP to ensure abnormal motor findings are not transient and watch to determine whether they will resolve [39]. As such, it is possible that infants in our sample not yet diagnosed with CP to date may be diagnosed later in life. Thus, we cannot yet reliably report on the outcome or characteristics of our sample as relates to CP diagnosis.

## 5. Conclusions

Absence of TMS elicited MEPs from the more-affected hemisphere was found to be related to corrected age and may be associated with atypical movement in infants with perinatal stroke at risk of cerebral palsy. TMS assessments could promote early identification of biomarkers of atypical corticospinal tract development within the first year of life. Longitudinal TMS assessments of infants with perinatal stroke could inform whether MEPs from the more-affected hemisphere are present early in infancy and lost later in development. Further investigations could identify a sensitive period of corticospinal tract development and guide optimal timing for intervention to maximize motor outcomes after perinatal stroke.

## Figures and Tables

**Table 1:** Infant clinical characteristics

Infant	Sex	GA (Weeks)	Level of Prematurity	CA at TMS (Months)	Neuroradiology Findings	Motor Outcome
1	M	31	Very preterm	3	Bilateral parieto-occipital cystic periventricular leukomalacia	Atypical (GMA)
2	M	41	Term	4	Left frontal lobe intraparenchymal hemorrhage with adjacent subdural and subarachnoid hemorrhage	Typical (GMA)
3	M	22	Extremely preterm	4	Grade 2 germinal matrix hemorrhage and multifocal cerebellar hemorrhage	Typical (GMA)
4	M	26	Extremely preterm	5	Bilateral cystic periventricular leukomalacia, ex vacuo dilatation of the lateral and third ventricles; left thinning of parietal and occipital lobes	Atypical (GMA)
5	F	26	Extremely preterm	6	Right grade III and left grade II intraventricular hemorrhage	Typical (GMA)
6	F	25	Extremely preterm	7	Bilateral cerebellar hemorrhages	Atypical (BSID-III)
7	F	22	Extremely preterm	9	Bilateral cerebellar hemorrhages, ex vacuo dilatation of the fourth ventricle	Atypical (BSID-III)
8	M	39	Term	10	Extensive bilateral ischemia in the right parietal, occipital, temporal, and posterior frontal lobes; basal ganglia and thalamus; and in left parietal, posterior temporal, posterior occipital, and frontal lobes	Atypical (BSID-III)
9	M	36	Moderate to late preterm	12	Left parietal encephalomalacic cleft, similar but milder cleft on right parietal vertex; hemosiderin deposits in left temporal, posterior left frontal, left parietal lobes	Typical (BSID-III)
10	M	41	Term	12	Gliosis and encephalomalacia along the right paracentral region, corona radiata, centrum semiovale, and posterior limb of internal capsule	Atypical (BSID-III)

GA: gestational age; CA: corrected age; GMA: Prechtl General Movements Assessment; BSID-III: Bayley Scales of Infant and Toddler Development III.

**Table 2:** Presence of MEPs from TMS of each hemisphere by corrected and gestational age groups

Hemisphere Stimulated	Corrected Age at TMS Assessment		Gestational Age	
	Younger Infants (3–6 Months CA)	Older Infants (7–12 Months CA)	Preterm (GA < 27 Weeks)	Term (GA ≥ 27 Weeks)
	*n* = 5	*n* = 5	*n* = 7	*n* = 3
**MAH** **LAH**	80%	0%	43%	33%
	100%	60%	86%	67%

Percentage of infants with a present motor evoked potential (MEP) from the more-affected hemisphere (MAH) or less-affected hemisphere (LAH). CA: corrected age for prematurity at time of transcranial magnetic stimulation (TMS) assessment; GA: gestational age.

**Table 3:** Individual infant MEP responses to transcranial magnetic stimulation

Infant	CA	MEP from MAH	MAH MSO	MEP from LAH	LAH MSO
1	3	Contralateral	80%	Contralateral	75%
2	4	Contralateral and ipsilateral	80%	Ipsilateral	70%
3	4	Contralateral and ipsilateral	85%	Contralateral and ipsilateral	80%
4	5	Absent	-	Contralateral and ipsilateral	70%
5	6	Contralateral	75%	Ipsilateral	85%
6	7	Absent	-	Contralateral and ipsilateral	70%
7	9	Absent	-	Absent	-
8	10	Absent	-	Absent	-
9	12	Absent	-	Contralateral	80%
10	12	Absent	-	Contralateral	85%

Motor evoked potentials (MEPs) are reported as being present in either contralateral and/or ipsilateral wrist flexors or absent. Maximum stimulator output (MSO) is reported as the lowest intensity at which an MEP was elicited from the more or less-affected hemispheres (MAH; LAH). Infants are ordered by corrected age (CA) youngest to oldest. Dotted line demarcates younger (infants 1–5) from older (infants 6–10) infant groups.

**Table 4:** Atypical movement across MEP outcome from TMS of each hemisphere

Motor Outcome	TMS Responses			
	Present MEP from MAH	Absent MEP from MAH	Present MEP from LAH	Absent MEP from LAH
	*n* = 4	*n* = 6	*n* = 8	*n* = 2
Atypical movement	25%	83%	63%	50%

Data reported as percentage of infants with atypical movement for each transcranial magnetic stimulation (TMS) response category. MEP: motor evoked potential; MAH: more-affected hemisphere; LAH: less-affected hemisphere.

## Supplementary Materials

The following are available online at https://www.mdpi.com/2077-0383/8/8/1208/s1, Figure S1: Pre-Stimulus and MEP AUC for all +MEP Trials; Table S1: Mean pre-stimulus and MEP AUC for all infants; Figure S2: Pre-Stimulus AUC Differences for −MEP and +MEP Trials.
