# Supplementary material for: Motor Evoked Potentials as Potential Biomarkers of Early Atypical Corticospinal Tract Development in Infants with Perinatal Stroke
Source: J Clin Med. 2019 Aug 13;8(8):1208. doi: 10.3390/jcm8081208 (PMC6723226; doi:10.3390/jcm8081208)
Supplement: Supplementary file 1 [file jcm-08-01208-s001.zip › jcm-552717-SI.pdf]

Supplementary Materials

S.1. Area Under the Curve Analysis

Descriptive statistics of mean and range AUC are reported. The area under the curve (AUC) for motor evoked potentials (MEP) and for the pre-stimulus electromyographic activity (−13 to −3 ms) of trials producing (+MEP) and not producing (−MEP) MEPs varied for each infant. The overall mean MEP AUC was 6,217 µV·ms (range = 950–50,773 µV·ms) and the mean pre-stimulus AUC for +MEP trials was 1,630 µV·ms (range = 472–8,408 µV·ms) (Figure S1, Table S1). Each infant's mean pre-stimulus AUC for +MEP trials were subtracted from the mean pre-stimulus AUC for −MEP trials, and the absolute value of the difference was calculated (Figure S2). The mean pre-stimulus AUC was lower in +MEP trials compared to −MEP trials in 5/8 infants (Table S1, Figure S2).

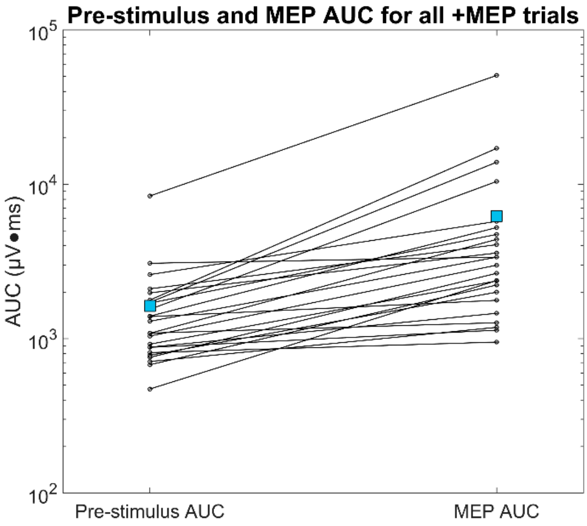

Figure S1. Area under the curve (AUC) for pre-stimulus and motor evoked potentials (MEP) for all trials in which an MEP was present (+MEP) reported on a log scale. Blue squares represent the mean pre-stimulus and MEP AUC for all +MEP trials.

Table S1. Mean pre-stimulus and MEP AUC for all infants.

Infant	Mean Pre-Stimulus AUC (µV·ms)		Mean MEP AUC (µV·ms)
	-MEP Trials	+MEP Trials	MEP
1	3,028	1,004	2,279
2	1,666	794	1,550
3	932	1,168	5,231
4	5,501	3,472	21,350
5	6,700	745	3,003
6	1,510	901	2,153
7	2,288	-	-
8	4,022	-	-
9	1,427	3,080	3,376
10	650	1,115	2,071

Mean area under the curve (AUC) for each infant's motor evoked potentials (MEP) and pre-stimulus activity for trials producing (+MEP) and not producing (−MEP) MEPs. AUC values for infants who did not demonstrate MEPs/+MEP trials are represented by dashes (−).

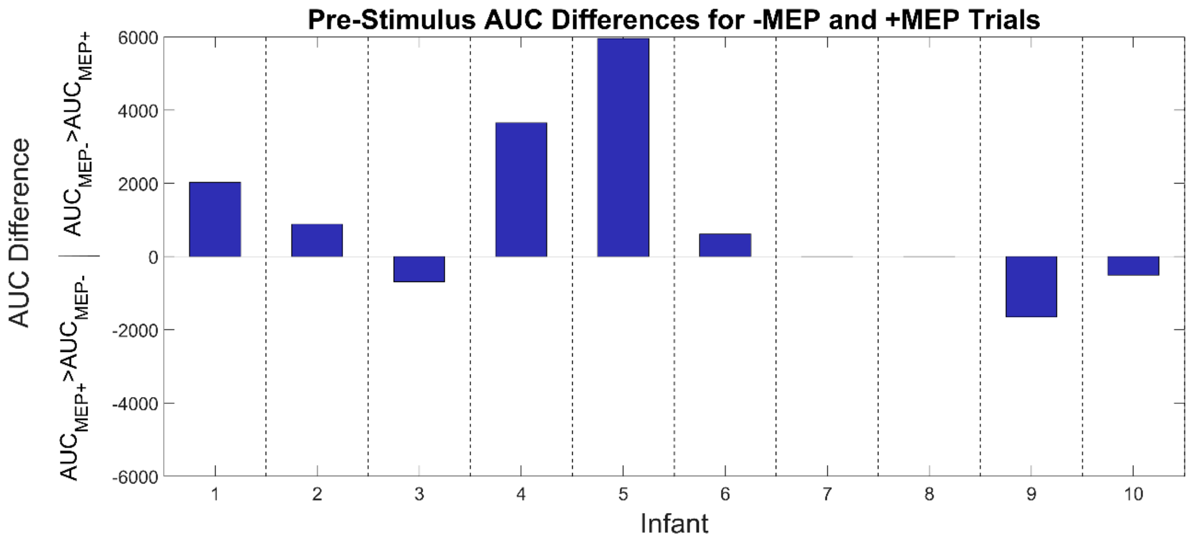

Figure S2. Mean difference of the pre-stimulus area under the curve (AUC) for trials producing motor evoked potentials (+MEP) and not producing motor evoked potentials (−MEP). Blue bars depict the absolute value of the difference between the mean pre-stimulus AUC for −MEP and +MEP trials for each infant. Bars in the upper quadrant represent −MEP pre-stimulus AUC that exceeded +MEP pre-stimulus AUC. Bars in the lower quadrant represent +MEP pre-stimulus AUC that exceeded −MEP pre-stimulus AUC. Bars are not present for infants 7 and 8 as no MEPs were elicited from these two infants.
